# Estimating the Optimum Coverage and Quality of Amplicon Sequencing With Taylor’s Power Law Extensions

**DOI:** 10.3389/fbioe.2020.00372

**Published:** 2020-05-15

**Authors:** Zhanshan (Sam) Ma

**Affiliations:** ^1^Computational Biology and Medical Ecology Lab, State Key Lab of Genetic Resources and Evolution, Kunming Institute of Zoology, Chinese Academy of Sciences, Kunming, China; ^2^Center for Excellence in Animal Evolution and Genetics, Chinese Academy of Sciences, Kunming, China

**Keywords:** Taylor’s power law (TPL), Taylor’s power law extension (TPLE), minimum sequencing reads (MSR), optimum sample size, amplicon sequencing, metagenetic sequencing

## Abstract

Theoretical analysis of DNA sequencing coverage problem has been investigated with complex mathematical models such as Lander–Waterman expectation theory and Stevens’ theorem for randomly covering a domain. In the field of metagenomics sequencing, several approaches have been developed to estimate the coverage of whole-genome shotgun sequencing, but surprisingly few studies addressed the coverage problem for marker-gene amplicon sequencing, for which arguably the biggest challenge is the complexity or heterogeneity of microbial communities. Overall, much of the practice still relies variously on speculation, semi-empirical and *ad hoc* heuristic models. Conservatively raising coverage may ensure the success of sequencing project, but often with unduly cost. In this study, we borrow the principles and approaches of optimum sampling methodology originated in applied entomology, achieved equal success in plant pathology and parasitology, and plays a critical role in the decision-making for global crop and forest protection against economic pests since 1970s when the pesticide crisis and food safety concerns forced the reduction of pesticide usages, which in turn requires reliable sampling techniques for monitoring pest populations. We realized that sequencing coverage is essentially an optimum sampling problem. Perhaps the only essential difference between sampling insects and sampling microbiome is the “instrument” used. In traditional entomology, it is usually humans that visually count the numbers of insects, occasionally aided by binocular microscope. In the metagenomics research, it is the DNA sequencers that count the number of DNA reads. Furthermore, a key theoretical foundation for sampling insect pest populations, i.e., Taylor’s power law, which achieved rare status of ecological law and captures the *population* aggregation, has been recently extended to the *community* level for describing community heterogeneity and stability, namely, Taylor’s power law extensions (TPLEs). This theoretical advance enabled us to develop a novel approach to assessing the quality and determining optimum reads (coverage) of amplicon sequencing operations. Specifically, two applications were developed: one is, in hindsight, to assess the quality of amplicon sequencing operation in terms of the *precision* and *confidence* levels. Another is, prior to sequencing operation, to determine the minimum sequencing efforts for a sequencing project to achieve preset *precision* and *confidence* levels.

## Introduction

Microbiome researchers employ two types of DNA sequencing technologies. One is the whole-genome shotgun sequencing (also known as metagenomic sequencing), and another is the marker gene (e.g., 16S-rRNA for bacteria or 18s-rRNA for fungi) amplicon sequencing. Existing approaches to studying the sequencing coverage problem for microbiome research have been focused on the former type, and surprisingly few studies have been on the amplicon sequencing.

Rodriguez-R and Konstantinidis (2014) first distinguished two terms in microbiome research, sequencing *coverage* (the fraction of the metagenome represented in the metagenomic dataset) vs. sequencing *depth* (repetition of features, which we are not concerned in this study). The significance of coverage problem is obvious. In extreme cases, when small datasets from sequencing with insufficient coverage are utilized to describe complex communities, statistical inferences become unreliable and may even generate misleading conclusions (Rodriguez-R and Konstantinidis, 2014). The coverage-size (data size of sequencing reads) curve is usually logistic-shaped and approaches saturation level when the sequencing efforts are sufficiently large. But, as rightly pointed out by Rodriguez-R and Konstantinidis (2014), coverage is not simply a function of dataset size. Instead, the relationship heavily depends on the *complexity* (i.e., *heterogeneity*) of the microbial communities sampled. Wendl et al. (2013) characterized current metagenomic project designs as relying on variously on speculation, semi-empirical and *ad hoc* heuristic models such as elementary extensions of single-sample Lander–Waterman expectation theory.

Existing approaches to investigating the sequencing coverage problem in metagenome research (some of the approaches are also applicable to marker gene, e.g., 16s-rRNA amplicon sequencing) may be categorized as the following five kinds. One of the most widely used approaches is the rarefaction curve, which is based on the principle that the curve of any rarefied counts of a feature should reach plateau when the sampling efforts are close to saturation (e.g., Chao et al., 2014). Nonetheless, the effectiveness of rarefaction approach is strongly contingent on the quality of assembly (clustering in the case of 16s-rRNA) or references database or both. The rarefaction approach for 16s-rRNA data can also be problematic because their high sequence conservation frequently masks important levels of genetic and ecological differences among closely related species (Caro-Quintero and Konstantinidis, 2012; Rodriguez-R and Konstantinidis, 2014). A second approach is to evaluate the coverage of one or a few target species in the metagenomic dataset using simple mathematical methods such as the Lander–Waterman formulae (Lander and Waterman, 1988; Wendl et al., 2013), but ignoring the rest genomes in the community. A disadvantage of this approach is the requirement of reliable estimates for genome sizes and the abundance of the targeted species, which usually poorly represents the community as a whole. A third approach is to use genome-wide approaches that capitalize on community modeling and/or modeling of contig sequencing depth (e.g., Hooper et al., 2010; Stanhope, 2010). A fourth approach is the redundancy-based approach, termed Non-pareil and developed by Rodriguez-R and Konstantinidis (2013, 2014). The approach is independent of assembly, reference databases or abundance distribution models, and can be used to compare different datasets. Non-pareil can project the average coverage at larger sequencing efforts, and estimate the amount of sequencing efforts needed to reach any given coverage level. The non-pareil estimates are made from the organisms recovered in a metagenomic data set and are abundance-weighted. The estimates preferentially represent the abundant organisms in a sample. If the goal is to characterize all members of the community, or rare members preferentially, the non-pareil estimates may be limited, and should be complemented with genome- or marker-based estimations (Rodriguez-R and Konstantinidis, 2014). A fifth category of approaches is derived from some relatively mature methods in single-species genomics research (e.g., Hooper et al., 2010; Wendl et al., 2013).

Sequencing platforms-dependent factors such as non-uniform coverage associated with prevalent NGS technology (Chouvarine et al., 2016) and standard of operations (Sinha et al., 2015) may also influence the sequencing coverage. Remedies include mock microbial communities (Brooks et al., 2015), GC-bias adjustment and filtration and normalization techniques (Chouvarine et al., 2016), and small pilot studies assisted by rarefaction (Pollock et al., 2018).

One may have noticed that in previous review the definition for coverage was not strictly distinguished between amplicon sequencing and whole-genome sequencing. In fact, in much of the existing literature, it is implicitly assumed that the concept is applicable to both amplicon and whole-genome sequencing. However, a careful examination would suggest that some minor adaptations are needed to properly use the coverage concept. According to Rodriguez-R and Konstantinidis (2014), sequencing coverage would be the fraction of the metagenome represented in the metagenomic dataset. A natural adaptation of this definition could be the fraction of operational taxonomic units (OTUs) represented in the amplicon sequencing reads. Without this adaptation, the coverage concept would not make full sense, which might explain why few existing studies addressed the coverage problem for amplicon sequencing data. Nevertheless, our adaptation generates two new issues. One issue is that the OTU (abundance) tables are essentially the same as the species abundance tables in macro-ecology of insects, plants or animals, but they are less similar to gene abundance tables from the whole-genome shotgun sequencing. This is because, unless genes can be classified into so-termed MGS (metagenomic species) (actually only limited number of genes can be classified as MGSs with current bioinformatics algorithms) (Nielsen et al., 2014), their taxonomic identities cannot be determined with current sequencing technologies and bioinformatics analyses. The second issue is that OTU abundance tables (distribution) are highly heterogeneous and the statistical distribution follows the highly skewed distribution (particularly power law distribution), which require statistical estimation and inference methods different from commonly used Gaussian-distribution-based methods. The second issue, of course, is due to the enormous heterogeneities of microbial communities, which is a well-known fact thanks to the HMP/MetaHIT and recent studies (Ehrlich and MetaHIT Consortium, 2010; HMP Consortium, 2012; The Integrative HMP (iHMP) Research Network Consortium, 2019).

The above two issues have been faced by entomologists, plant pathologists and parasitologists at least since 1970s when pesticide crisis and consequent public concerns on food safety and environmental pollution prompted the search for alternatives to pesticides such as biological control (which relies on natural enemies). Strategically, the pesticides crisis, as vividly described by Carson (1962) in her now classic “Silent Spring,” also forced the adoption of so-called integrated pest management (IPM), the first principle of which is to tolerate pest (including insects, plant pathogens and nematodes) unless the pest population size crossed the so-termed “economic tolerance threshold.” Both IPM and biological control obviously require reliable monitoring of pest population (*relative*) abundances by sampling techniques (estimation of total or *absolute* abundances is neither possible nor necessary). A theoretical foundation for pest-sampling technique is Taylor’s power law (TPL), which achieved somewhat rare status of *law* in ecology (Taylor, 1961, 1984, 2019; Taylor and Taylor, 1977; Taylor et al., 1983, 1988). Essentially, TPL established an extremely robust power relationship between population abundance (*m*) and its variance (*V*), which has been verified by hundreds (if not thousands) of field observations in various organisms. In fact, the TPL has been found to exist in many fields beyond entomology and ecology including computer science and molecular biology (Ma, 2012, 2015; Li and Ma, 2019a), which are comprehensively reviewed and synthesized in a recent monograph by Taylor (2019). This *V-m* power law relationship bridges a gap between sampling biological populations and rigorous *optimum sampling theory* in statistics. For the above-described reasons, TPL-based optimum sampling techniques have become widely adopted in global crop/forest protection against pests as a key technique of the IPM (e.g., Ifoulis and Savopoulou-Soultani, 2006; Jovani and Tella, 2006; Wright and Palukkatu-Thodi, 2016; Shvydka et al., 2018). It was also applied to fishery and water quality monitoring where sampling estimations and monitoring of organisms abundances are necessary. For example, The EU’s standard “EVS-EN14757L2005” was established for water quality sampling in fishery management with multi-mesh gillnets (European Standard, 2005). These experiences in sampling macrobial organisms (such as insect pests) are valuable for us to tackle the problem of optimum sequencing reads because the fundamental mathematical model (i.e., TPL model) and statistical methods (i.e., for determining the minimum sample sizes or sampling efforts) can be translated into a solution for sampling microorganisms, as demonstrated in later sections. In fact, the same technique has already been applied to microbial sampling for the damages of parasites and/or plant pathogens as briefly reviewed previously since 1970s. The only essential difference between previous sampling experiences and the scenario of this study is the “instrument” for counting the organisms or their “proxy.” In previous experiences, visually counting insects by humans or microscope for counting plant pathogens can be the means (“instruments”), and in the present article, DNA sequencers counting the DNA reads are the instruments.

For 16S-rRNA amplicon sequencing, the mission is to obtain sufficient number of high quality reads that can be mapped to bacterial species (or OTUs). If the number of 16s-rRNA reads is not sufficiently large, low abundance species in the microbial community may not be detected. More seriously, properties of the sampled community may be wrongly characterized. But if the number is too large or rare species are not a concern, obtaining excessively large number of reads can be wasteful economically. This mission is essentially the same as sampling sufficient number of sampling units to detect the abundance of pests (insects, plant pathogens, nematodes, or parasites). This is why, in this study, we choose to learn from the successes of TPL-based optimum sampling in applied entomology, plant pathology and parasitology. As explained previously, we need to make slight adaptation to the concept of coverage for amplicon sequencing. In consideration of its conventional usage and compatibility with the mission of 16S-rRNA sequencing, we use the term *coverage* loosely referring to the number of sequencing reads of 16s-rRNA or other marker genes (such as 18s-rRNA) obtained from amplicon or other metagenetic sequencing operations. We could have adopted a direct adaptation of Rodriguez-R and Konstantinidis (2014)—the fraction of OTUs represented in the amplicon sequencing reads—as explained previously, but the direct adaptation is not convenient for linking with the TPL-based optimum sampling approach from economic entomology. Obviously, sequencing coverage has an implicit and innate aspect of quality control; we introduce two additional statistics, i.e., confidence level (*p*-value) and precision level to ensure our loosely defined coverage concept can take advantages of the TPL-based optimum sampling methodology. In addition, we use the terms *minimum* reads and *optimum* reads interchangeably with an implicit assumption, i.e., the optimum is the minimum in consideration of both sequencing quality and cost.

When applying TPL-based optimum sampling technique to address the coverage problem of amplicon sequencing, a new (third) issue regarding the sampling entity (target) occurs. The entity of microbiome sequencing (sampling) is community but that of insect sampling is population. In a previous study (Ma, 2015), we had extended TPL to the community level and tested it with the HMP datasets, which removed the last roadblock for introducing the sampling technique from economic entomology to estimating the optimum coverage and quality of amplicon sequencing.

**FIGURE 1:**
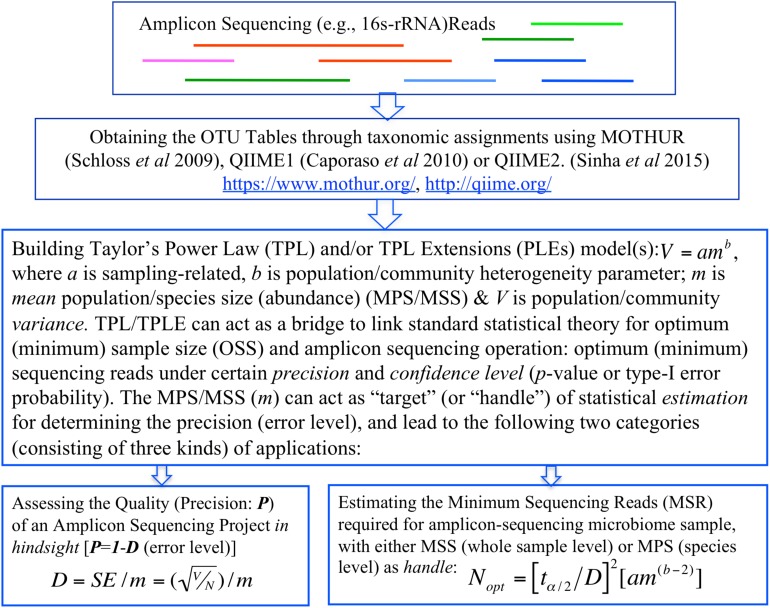
A diagram showing the procedures (steps) to implement the proposed TPP/TPLE-based optimum sample size approach for: (i) assessing the quality of an amplicon-sequencing project *in hindsight*; (ii) estimating *minimum sequencing reads* designed for sequencing whole microbiome sample based on the TPLE; or (iii) estimating *minimum sequencing reads* designed for monitoring specific species based on the TPL.

In summary, the objective of this study is two-fold, corresponding to two categories of TPL/TPLE-based applications we introduce in this article. One is, in hindsight, to assess the quality of amplicon sequencing operation in terms of the *precision* and *confidence level* (*p*-value). Another is, prior to sequencing operation, to determine the minimum sequencing efforts (optimum sequencing coverage)—the minimum sequencing reads required to meet certain level of preset *precision* and *confidence level* (*p*-value) for sequencing a microbiome sample or for monitoring a specific species in a community sample (Figure 1).

## Methods

Taylor’s power law was first discovered in ecology by British ecologist Lionel Roy Taylor (1924–2007) (Taylor, 1961, 1984, Taylor and Taylor, 1977; Taylor et al., 1983, 1988) and by now has been validated by tens of hundreds field observations, not only in ecology, but also in many other fields of natural and social sciences. In recent years, TPL has again attracted renewed theoretical interests (e.g., Eisler et al., 2008; Cohen et al., 2012, 2016; Stumpf and Porter, 2012; Cohen and Xu, 2015; Giometto et al., 2015; Lagrue et al., 2015; Ma, 2015; Xu, 2016; Xu et al., 2016; Reuman et al., 2017), and a recent monograph (Taylor, 2019) reviewed and synthesized the field timely and comprehensively.

The TPL It has a mathematical form,

(1) $$V=a\,m^{b}$$

where *a* and *b* are parameters from fitting the TPL with pairs of mean (*m*) and variance (*V*) obtained from sampling biological populations through time, space or both. The traditional biological entities of TPL were limited to the population level (Taylor, 1961, 1984; Taylor and Taylor, 1977; Taylor et al., 1983, 1988). It was extended to the community level by Ma (2015) with four extensions (Taylor’s power law extensions or TPLEs), i.e., Type-I TPLE for measuring the *community spatial heterogeneity*, Type-II TPLE for *community temporal stability*, Type-III TPLE for *mixed-species spatial heterogeneity* (*aggregation*), and Type-IV TPLE for *mixed-species temporal stability* (Ma, 2015).

Both the TPL and TPLEs have the same mathematical form, i.e., Eq. 1, but the interpretations of variables (*m*, *V*) and parameters (*a* and *b*) are different at population and community scales. Parameter *b* is a species-specific (the traditional TPL) or community-specific (the TPLEs), but parameter *a* is strongly influenced by sampling approaches, and is a function of sampling efficiency (Taylor et al., 1998; Ma, 2015; Taylor, 2018). The mean (*m*) in the traditional TPL refers to the *mean population size* (abundance) (MPS) per spatial or temporal sampling unit, and *V* is the corresponding variance at the population scale. The mean (*m*) in the Type-I and Type-II TPLE refers to the *mean species size* (abundance) (MSS) *per species*, and *V* is the corresponding variance at the community scale. Both MPS and MSS are *relative* abundance (size) since they are measured or estimated per *sample* or per *species* (strictly per sample per species). For example, assuming three species A, B, and C with per-sample abundance (size) of 2, 4, and 6 respectively, then the MSS for the three-species community is equal to 4. Estimating the absolute or total species (population) abundances is neither feasible nor necessary in most cases. For the fitting of TPL and TPLEs and their interpretations, readers are referred to Taylor (1961, 1984) and Ma (2015), respectively.

TPL and its extensions TPLEs have been found to characterize the *m*–*V* relationship universally well, which leads to the recognition of TPL as one of few classic laws in theoretical ecology (Taylor, 2019). In applied ecology, the most important application of TPL turned out to be in sampling design for estimating population abundance. This is because, as explained below, the *m*–*V* relationship is necessary for computing minimum sample size (sequencing reads in our case), also known as the optimum sample size in consideration of the cost-saving with minimum sampling efforts.

The analytic approach to determining an optimum sample size (*N*) (also known as the optimum number of sampling units), or the optimum (or minimum) sequencing reads in our case, for estimating the mean population abundance is based on the general formula by Karandinos (1976),

(2) $$N_{o\,p\,t}={\left(\frac{t_{\alpha /2}}{D}\right)}^{2}\,\left(\frac{V}{m^{2}}\right),$$

where *t*_*α/2*_ is Student’s *t*-distribution value such that *P*(*t* > *t*_α_) = α/2. *D* is actually used to define half-width of the confidence interval as a fixed proportion of the mean. For a 95% confidence interval, α = 0.05, *t*_α/2_ = 1.96≈2. In Eq. 2, *V* and *m* are the variance and mean of population abundance, respectively. Eq. 2 is based on general sampling theory in elementary statistics and it seemed that Karandinos (1976) was the first who introduced the approach to entomology.

Plug TPL model Eq. 1 into Eq. 2, the optimum sample size can be estimated with the following formula:

(3) $$N_{o\,p\,t}={\left(\frac{t_{\alpha /2}}{D}\right)}^{2}\,a\,m^{\left(b-2\right)}$$

where *a* and *b* are the parameter from TPL or TPLE (the different implications are explained below), *t*_*α/2*_ is Student’s *t*-distribution value and depends on the number of sampling units and approximates to *2* for more than 10 samples at the 95% level of confidence (*p* = 0.05). Several authors independently derived the above Eq. 3 (e.g., Ma, 1988, 1989, 1990; Duncan et al., 1989), and the approach is not only widely adopted in entomology and insect pest management, but also in other fields where estimating and monitoring the abundance of organisms are necessary, such as sampling and monitoring nematode, parasites, plant pathogens, fishery, and water quality monitoring (e.g., European Union’s standard “EVS-EN14757L2005”; European Standard, 2005; Xu et al., 2016). For another example, The US Forest Service’s decision support software for monitoring Gypsy moth also used TPL-based sampling approach (Taylor et al., 1991; 2018).

In Eqs 2 and 3, *D* is the error level, and defined as the standard error of the mean (*SE*) (i.e., standard error per unit of mean or the standard error divided by the mean). The *SE* is equal to standard deviation ($s=\sqrt{V}$) divided by the square root of sample size ($\sqrt{N}$), that is,

(4) $$D=S\,E/m=\left(s/\sqrt{N}\right)/m=C\,V/\sqrt{N}$$

where *CV* is the coefficient of variation and is equal to $s/m=\sqrt{V}/m$.

*D* is also known as *allowable error* or *fixed precision* level, with which the *mean* (*m*) is measured. For example, *D* = 0.30 or 30% represents that the *sample mean* may be 30% higher or lower than the *population* (*sensu* statistics or actual) *mean* in 95% of the occasions (e.g., sampling is repeated 100 times) (95% confidence limits). *P* = 1 - *D* is often termed as *relative precision*. For example, when *D* = 0.3, *P* = 1 - 0.3 = 0.70 = 70%, one can say that the *relative precision* for this sampling operation is 70% and the precision can be achieved in 95% of times. In other words, there are 5% of times when the pre-specified precision level of 70% may not be reached.

As further classified and demonstrated in the next section, we introduce two major categories of sampling applications. One category is based on the TPLE at the *community* level, specifically Type-I TPLE for community spatial heterogeneity or Type-II TPLE for community temporal stability. As a side note, Type-III and Type-IV TPLEs are built for the mixed species and may not be suitable for sampling design. Another category is based on the traditional TPL or what we term as single-species power law, given that the TPL is constructed at the population (*sensu* biologically) level.

The second category (TPL-based) of applications is essentially the same with the applications widely adopted in applied entomology and IPM decision-making as well as other fields mentioned previously, and they are designed for monitoring (by sampling) the abundance of a specific species (or OTU). In this category, the TPL parameters (*a* and *b*) for single-species population are plugged into Eq. 3 to compute corresponding optimum sample size (*N*) under a certain relative precision (*P* = 1 − *D*) and confidence level. In the case of the 16s-rRNA sequencing coverage problem, the optimum sample size (*N*) computed from Eq. 3 corresponds to the minimum sequencing reads required for estimating the abundance of a specific species or OTU.

The first category of applications is based on the TPLEs (Ma, 2015). This is a new application of the optimum sample size formula tailored for *community* (or microbiota) level. In this category, the parameters (*a* and *b*) from Type-I or Type-II TPLE are plugged into Eq. 3 to compute the optimum sample size under a certain relative precision level and confidence level. In the case of 16s-rRNA sequencing based coverage problem addressed in this paper, the optimum sample size (*N*) corresponds to the minimal sequencing reads required to estimate mean species size (abundances) (MSS) *per* species in a microbiome sample reliably and confidently (specified by relative precision and confidence level). The difference between Type-I TPLE and Type-II TPLE based sampling scheme lies in space vs. time. The spatial version Type-I TPLE is constructed to measure the spatial heterogeneity (e.g., inter-individual) based on the cross-sectional data of many individuals (or habitats in general), and in contrast, the temporal version Type-II TPLE is constructed based on the longitudinal data of one individual (or one piece of habitat in general). Therefore, the optimum sample size based on Type-I TPLE is suitable for cross-sectional sampling, and that based on Type-II TPLE suitable for longitudinal sampling.

Regarding the TPLE based optimum sample sizes for addressing 16s-rRNA sequencing coverage problem, there are two additional important intricacies, which we briefly described here, but the detailed discussion is deferred to the next section with illustrative examples. In general, there are two ways to apply the optimum sample size formula (Eq. 3) at the community level. One approach is similar to the application based on the traditional TPL, i.e., for estimating the MSS (i.e., the total abundances of all species divided by the total number of species in the sampled community) under a pre-specified relative precision and confidence level. An alternative approach is to harness the power of the formula for “reversely” assessing the quality of a sequencing project, because if we know the MSS (which is known after the completion of sequencing), we can compute the relative precision of the sequencing operation. We will demonstrate both the applications in the next section. Figure 1 illustrates the steps (procedures) to implement the proposed method for assessing the sequencing *quality* or estimating the *optimum sequencing reads* (minimum sequencing efforts) for amplicon sequencing based on TPL/TPLE model parameters.

## Demonstrations

### The 16S-rRNA Datasets for Demonstration

We use two datasets to demonstrate the applications of TPL/TPLE based optimum sample size formula. The first dataset is from the American Gut Project (AGP)^1^, part of the Earth Microbiome Project (EMP), and is co-founded by Dr. Rob Knight and Dr. Jeff Leach at the University of California, San Diego. The AGP OTU tables were rarefied to 10,000 sequence reads per sample and computed from the DNA-sequencing data of 16s-rRNA (v4 region) marker genes from the gut microbiome of 6500 volunteer participants (as of October 2015), and downloaded from the AGP website^2^. We selected the dataset of 1473 healthy Caucasian individuals and excluded the samples from individuals with IBD, diabetes and any other diseases. The cross-sectional AGP dataset is utilized to build Type-I TPLE models for demonstrating the optimum sequencing reads in a cross-sectional study.

The second dataset we utilized is from a longitudinal study on the HVM (human vaginal microbiome) by Gajer et al. (2012) sampled from 32 healthy women at reproductive age. The dataset is one of the longest and also the most comprehensive longitudinal study of microbiome dynamics, conducted with 16*s*-rRNA amplicon sequencing technology. We term this dataset 32-cohort HVM (Human Vaginal Microbiome) dataset hereafter. This dataset is utilized to build Type-II TPLE models for demonstrating the optimum sequencing reads in a longitudinal study.

### Building Type-I and II TPLE Models

To demonstrate the utilizations of the TPLE-based optimum sample size, we first need to obtain the parameters (*a* and *b*) of power law extensions (TPLEs) and then plug the acquired parameters into Eq. 3 for computing the minimum (optimum) reads. Table 1 below listed the Type-I TPLE model parameter values fitted to the AGP (American Gut Project). The parameter *b* of TPLE should be community (microbiome) specific, and should be invariant of sampling environment such as sequencing platform (Ma, 2015). In contrast, the parameter *a* of TPLE is not community (microbiome) specific and may vary between different sequencing platforms. In fact, parameter *a* may be strongly influenced by environmental and sampling factors (Taylor, 1961, 1984, Taylor and Taylor, 1977; Ma, 2015). The variability of TPLE parameter *a* also means that estimating the optimum sample size is influenced by sampling procedure (which is captured by parameter *a*) besides the type of microbiome (which is captured by parameter *b*), pre-specified precision and confidence levels (*D*, *t*_α/2_), which is also evident from Eq. 3. All parameters but the parameter *a* are controllable in the sense that only parameter *a* is specific to a sampling operation or to a specific sequencing operation in the case of this study. This is a limitation of any sampling procedure but also a reality, which remind us that the sequencing coverage problem is also dependent on sequencing platform. The intricacy that the parameter (*a*) of TPLE or TPL can capture the sequencing platform specificity should actually be an advantage or flexibility of our proposed approach.

**TABLE 1:** Type-I TPLE (Taylor’s Power Law Extension) parameters for the AGP (American Gut Project).

Datasets	*b*	ln(*a*)	CACD	*R*	*p*	*N*
AGP	1.831	6.636	0.0003	0.797	0.000	1473

Type-II TPLE for community temporal stability can also be utilized for constructing the optimum sample size formula at the community level, but for monitoring the temporal changes of a community in a longitudinal setting. In this study, we demonstrate the temporal application with the Type-II TPLE models previously built for the 32-healthy cohort originally designed for investigating the temporal dynamics of the human vaginal microbiome (HVM) of 32 healthy women (Gajer et al., 2012; Ma, 2015). The relevant parameters (*a* and *b*) of Type-II TPLE for the 32-healthy cohort were excerpted from our previous publication (Ma, 2015) as Supplementary Table S7 in the Online Supplementary Information (OSI), but not listed in the main text of this article.

### Assessing the *Quality of Amplicon Sequencing* Operations *in Hindsight* Based on Type-I or Type-II TPLE

As stated in previous section, there are two promising applications with the TPLE-based optimum sample size, we demonstrate its first application—evaluating the quality of a finished sequencing operation—here, and its second application—computing the optimum coverage (minimum sequencing reads) under pre-specified relative precision and confidence levels—in the next sub-section.

In this venue, the MSS *per* species is known after finishing the sequencing operation and necessary bioinformatics analysis, which can be directly computed as the total sequencing reads (abundances) of all species detected divided by the total number of species detected. The number of total reads from the sequencing operation is also known. We can easily calculate the relative precision *P* = (1 - *D*) with Eq. 3. Obviously, *P* can be a quality measure of the sequencing operation.

The bottom section of Table 2 shows that the actual relative precision levels (*P*) computed for the AGP is 66%. This estimation of precision of the AGP project should be rather reasonable, and it also demonstrated the feasibility of the proposed approach. As a side note, the top section of Table 2 listed the table of minimum sequencing reads corresponding to various levels of relative precision levels, i.e., the demonstration of another potential application of the optimum sample size explained in the next sub-section.

**TABLE 2:** The minimum sequencing reads required to achieve certain *precision* (*P*) levels estimated with Type-I TPLE-based optimum sample size formula for the AGP, as well as the *actual* precision level of the AGP (the bottom section) estimated *in hindsight*.

*D* (Error Level)	*P* = (*1 − D*) (Precision)	AGP: Mean Species Size (*per* Species) = 2.162
		Minimum Reads
0.10	0.90	267,566
0.15	0.85	118,918
0.20	0.80	66,891
0.25	0.75	42,811
0.30	0.70	29,730
0.35	0.65	21,842
0.40	0.60	16,723
0.45	0.55	13,213
0.50	0.50	10,703
The *Actual Relative Precision* of the AGP datasets (MSS = 2.162)		
0.34	*0.66*	23,634

Similarly, we can evaluate the sequencing quality of the 32-healthy cohort project in terms of the relative precision in estimating the MSS for each of the 32 subjects in the 32-healthy cohort. Table 3 below shows the results of the three subjects (#400, #430, and #439) excerpted from Supplementary Table S1 in the OSI (Online Supplementary Information), where the complete results for all 32 subjects were exhibited. The relative precision levels of the 32-helathy cohort ranged from 58–90% with an average of 76%.

**TABLE 3:** The sequencing *precision* “reversely” estimated *in hindsight* with TPLE-based optimum sample size formula for the 32-cohort HVM (see Supplementary Table S1 for full results).

Subject ID	*M*	*Reads*	*D* (Error Level)	*P* = (1 – *D*) (Precision)
#400	43.383	2603	0.255	0.745
#430	49.133	4127	0.104	0.896
#439	55.327	2324	0.181	0.819
…	…	…	…	…
Average	39.613	2710	0.244	0.756

The major difference between Type-I TPLE and Type-II TPLE based sampling (Table 2 vs. Table 3) is that the former is applicable to cross-sectional study and the latter is applicable to longitudinal study of an individual’s microbiome. This is because Type-I TPLE captures the community spatial heterogeneity (variability) information, while Type-II TPLE captures the community temporal stability (variability) information.

### Estimating the Minimum Coverage Required for Amplicon-Sequencing Microbiome Sample (i.e., at Community Scale) Based on Type-I or II TPLE

**FIGURE 2:**
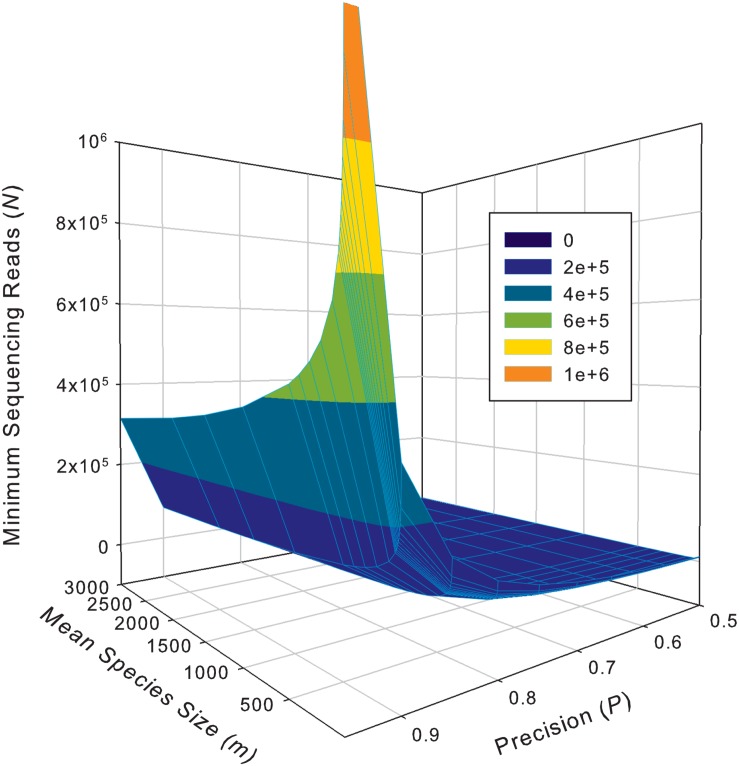
The 3D graph showing the minimum sequencing reads (coverage) required for achieving various pre-specified precision levels, computed with Type-I TPLE based optimum sampling formula.

While the previous application is essentially a hindsight evaluation of a sequencing operation, what we demonstrate below in Table 4 are foresight estimates of the minimum sequencing reads (i.e., the sequencing coverage loosely) required to achieve certain precision levels, under assumed MSS (mean species size, noted as *m*) per species. For example, for AGP sampling displayed in the top section of Table 4, if we assume precision *P* = 70% with confidence level of 95% (i.e., α = 0.05, the probability of committing a type-I error), the column corresponding to *P* = 0.70 (error level *D* = 0.3) listed the minimum sequencing reads required to meet the relative precision level of 70% under different MSSs ranging from *m* = 1 to 1000. We observed the decreasing number of minimum reads with the increase of the MSS (*m*), ranging from 33,868 to 10,539, corresponding to *m* = 1, 1000 respectively. This is reasonable because with higher *m*, less sampling efforts or fewer reads should be needed to reach a certain precision. Another trend in the top AGP section of Table 4 is that higher precision levels require more minimum reads. The trend is better illustrated in Figure 2.

**TABLE 4:** The minimum sequencing reads (MSR) required for characterizing the species composition and abundance of microbiome sample (i.e., at microbiome sample scale) under certain precision levels with preset confidence level (*p* = 0.05): the top section demonstrates the Type-I TPLE based optimum sample size formula with AGP datasets, and the bottom section demonstrate the Type-II TPLE based counterpart with 32-cohort HVM datasets.

*M*	*D* (Error Level) and *P* = (1 - *D*) (Precision Level)										
	*D* = 0.01	0.05	0.10	0.15	0.20	0.25	0.30	0.35	0.40	0.45	0.50
	*P* = 0.99	0.95	0.90	0.85	0.80	0.75	0.70	0.65	0.60	0.55	0.50
**Minimum sequencing reads table for the AGP based on Type-I TPLE**											
1	30,481,629	1,219,265	304,816	135,474	76,204	48,771	33,868	24,883	19,051	15,053	12,193
5	23,222,675	928,907	232,227	103,212	58,057	37,156	25,803	18,957	14,514	11,468	9289
10	20,655,617	826,225	206,556	91,803	51,639	33,049	22,951	16,862	12,910	10,200	8262
20	18,372,324	734,893	183,723	81,655	45,931	29,396	20,414	14,998	11,483	9073	7349
30	17,155,551	686,222	171,556	76,247	42,889	27,449	19,062	14,005	10,722	8472	6862
40	16,341,429	653,657	163,414	72,629	40,854	26,146	18,157	13,340	10,213	8070	6537
50	15,736,648	629,466	157,366	69,941	39,342	25,179	17,485	12,846	9835	7771	6295
100	13,997,103	559,884	139,971	62,209	34,993	22,395	15,552	11,426	8748	6912	5599
200	12,449,850	497,994	124,498	55,333	31,125	19,920	13,833	10,163	7781	6148	4980
300	11,625,314	465,013	116,253	51,668	29,063	18,601	12,917	9490	7266	5741	4650
400	11,073,631	442,945	110,736	49,216	27,684	17,718	12,304	9040	6921	5468	4429
500	10,663,806	426,552	106,638	47,395	26,660	17,062	11,849	8705	6665	5266	4266
1000	9,485,018	379,401	94,850	42,156	23,713	15,176	10,539	7743	5928	4684	3794
**Minimum sequencing reads table for the 32-cohort HVM based on Type-II TPLE**											
(Only results for two subjects are displayed and see Supplementary Table S2 for the full results of the whole cohort)											
**Subject #400**											
1	91,978	3679	920	409	230	147	102	75	57	45	37
5	319,116	12,765	3191	1418	798	511	355	261	199	158	128
10	545,289	21,812	5453	2424	1363	872	606	445	341	269	218
20	931,764	37,271	9318	4141	2329	1491	1035	761	582	460	373
30	1,274,718	50,989	12,747	5665	3187	2040	1416	1041	797	629	510
40	1,592,152	63,686	15,922	7076	3980	2547	1769	1300	995	786	637
50	1,891,866	75,675	18,919	8408	4730	3027	2102	1544	1182	934	757
60	2,178,176	87,127	21,782	9681	5445	3485	2420	1778	1361	1076	871
70	2,453,799	98,152	24,538	10,906	6134	3926	2726	2003	1534	1212	982
80	2,720,592	108,824	27,206	12,092	6801	4353	3023	2221	1700	1344	1088
90	2,979,898	119,196	29,799	13,244	7450	4768	3311	2433	1862	1472	1192
100	3,232,729	129,309	32,327	14,368	8082	5172	3592	2639	2020	1596	1293
**Subject #430**											
1	622,226	24,889	6222	2765	1556	996	691	508	389	307	249
5	542,889	21,716	5429	2413	1357	869	603	443	339	268	217
10	511,917	20,477	5119	2275	1280	819	569	418	320	253	205
20	482,712	19,308	4827	2145	1207	772	536	394	302	238	193
30	466,406	18,656	4664	2073	1166	746	518	381	292	230	187
40	455,172	18,207	4552	2023	1138	728	506	372	284	225	182
50	446,645	17,866	4466	1985	1117	715	496	365	279	221	179
60	439,797	17,592	4398	1955	1099	704	489	359	275	217	176
70	434,089	17,364	4341	1929	1085	695	482	354	271	214	174
80	429,204	17,168	4292	1908	1073	687	477	350	268	212	172
90	424,941	16,998	4249	1889	1062	680	472	347	266	210	170
100	421,164	16,847	4212	1872	1053	674	468	344	263	208	168

The table of estimated minimum reads from Type-II TPLE for the 32-healthy HCMC datasets, listed in the bottom section of Table 4 below, showed a similar pattern as in the case of Type-I TPLE for AGP datasets in the top section, except that one sub-table was listed for each individual in the 32-cohort. The bottom section of Table 4 listed the look-up tables for two subjects (#400, #430) in the 32-healthy HVM cohort and the full results for all subjects in the cohort were exhibited in Supplementary Table S2 (Supplementary Excel file) of the OSI.

An interesting phenomenon exhibited in Table 4 (the bottom section) for Type-II TPLE-based optimum sampling formula happened with subject #400. Specifically, for subject #400, estimating the higher MSS actually required more sampling efforts (reads). The underlying mechanism for this phenomenon is that the *b*-value of TPLE for subject #400 exceeded 2 (*b* = 2.773) as exhibited in Supplementary Table S7 or in Ma (2015). When *b* > 2, the heterogeneity (variability) is so higher that more sampling efforts are actually needed to reliably estimate the MSS. This example demonstrated the far-reaching influence of the heterogeneity (variability) on the sequencing coverage requirements, which is nicely captured by community specific TPLE parameter (*b*), an inherent advantage of our proposed approach.

### Estimating the Minimum Coverage Required for Monitoring *Single-Species* Abundance Based on the Classic TPL

While the previous sub-section demonstrated the computation of optimum sequencing reads at the whole microbiome sample level, in this sub-section, we demonstrate the estimation of optimum reads at individual species level. This application is based on the traditional TPL, and, in principle, has no differences with its application in applied entomology, plant pathology and parasitology. To apply the TPL-based sampling formula, we first need to construct the TPL model for each single species in the AGP or 32-cohort HMV datasets, respectively. For the AGP dataset, to build single-species TPL models, we divided 1473 samples into 26 groups (the large sample size statistically, and this is equivalent to divide each species into 26 population samples). For each species, we then computed the mean and variance for each group and obtained a total of 26 pairs of *V–m* pairs, fitted to TPL model with the *V–m* pairs, and obtained the TPL parameters. The TPL model parameters for all 2838 species were listed in Supplementary Table S3 (Supplementary Excel file) in the OSI, and the results for 10 selected species were excerpted from Supplementary Table S3 and listed in the top section of Supplementary Table S8. The 10 species were selected to represent the full spectrum of mean population abundance (per sample) in the AGP datasets because the optimum sample size to be estimated is strongly dependent on the population abundance. We divided the 2838 species into 10 intervals based on the order of mean population abundance and selected one species from each interval.

For the 32-cohort HVM datasets, we obtained the TPL parameters for single-species populations from our previous study (Ma, 2015) and excerpted them as OSI Supplementary Table S4. The 10 species in the bottom section of Supplementary Table S8 were selected from Supplementary Table S4 to represent the so-called type-indicator species for the HVM proposed by Ravel et al. (2011) and Gajer et al. (2012).

**FIGURE 3:**
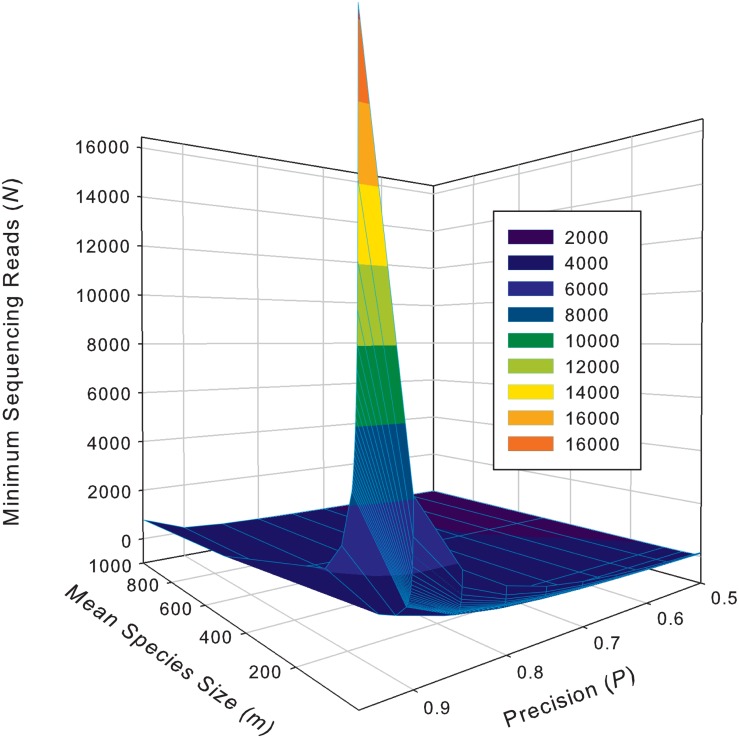
The 3D graph showing the minimum sequencing reads (coverage) for monitoring the community state type (CST) indicator species, *L. inners*, required for achieving various pre-specified precision levels, computed with single-species TPL-based optimum sampling formula.

Supplementary Table S5 exhibits the tables of the minimum sequencing reads for each of the 10 selected species (top section of Supplementary Table S8) from the AGP study, under different pre-defined precision levels. Supplementary Table S6 (Supplementary Excel file) exhibits the tables of the minimum sequencing reads for each of the species from the HVMC study (listed in Supplementary Table S4) under different pre-defined precision levels. Table 5 below is excerpted from Supplementary Table S6, and it contains the minimum sequencing reads table for species *Lactobacillus inners*. Figure 3 is the 3D graph showing the minimum sequencing reads (coverage) for monitoring the community state type (CST) indicator species, *L. inners*, required for achieving various pre-specified precision levels, computed with single-species TPL-based optimum sampling formula.

**TABLE 5:** The *minimum sequencing reads* table for species *L. inners*, required to achieve certain precision levels (*P*) for estimating the single-species population abundance (of *L. inners*) based on the TPL-based optimum sampling formula: excerpted from Supplementary Table S6 (containing the full results of all species listed in Supplementary Table S4).

*m*	*D* (Error level) or *P* = 1 - *D* (Precision)										
	*D* = 0.01	0.05	0.10	0.15	0.20	0.25	0.30	0.35	0.40	0.45	0.50
	*P* = 0.99	0.95	0.90	0.85	0.80	0.75	0.70	0.65	0.60	0.55	0.50
***L. iners***											
1	410,835	16,433	4108	1826	1027	657	456	335	257	203	164
2	302,585	12,103	3026	1345	756	484	336	247	189	149	121
3	253,019	10,121	2530	1125	633	405	281	207	158	125	101
4	222,858	8914	2229	990	557	357	248	182	139	110	89
5	201,962	8078	2020	898	505	323	224	165	126	100	81
6	186,352	7454	1864	828	466	298	207	152	116	92	75
7	174,099	6964	1741	774	435	279	193	142	109	86	70
8	164,138	6566	1641	730	410	263	182	134	103	81	66
9	155,826	6233	1558	693	390	249	173	127	97	77	62
10	148,748	5950	1487	661	372	238	165	121	93	73	59
20	109,555	4382	1096	487	274	175	122	89	68	54	44
50	73,123	2925	731	325	183	117	81	60	46	36	29
100	53,856	2154	539	239	135	86	60	44	34	27	22
500	26,475	1059	265	118	66	42	29	22	17	13	11
1000	19,499	780	195	87	49	31	22	16	12	10	8

The difference between the TPLE-based (the previous section) and traditional TPL-based (here) optimum sample size approaches lies in the scale of sampling operation. The TPLE-based optimum reads are designed for estimating the community-scale (microbiota or microbiome scale) MSS (*mean species size: M*), and therefore, it should be utilized to assess the quality of amplicon-sequencing from the perspective of microbiome sample. For individual species, the TPLE-based approach may offer little insights on the quality (precision) of their population abundance estimates. In contrast, the TPL-based approach is designed for estimating the MPS (*mean population size: m*) of single species and should be utilized to guide the sequencing design for monitoring (estimating) the abundance of individual species, which can be of particular biological significance such as community-type indicators (as defined by Ravel et al., 2011 and Gajer et al., 2012 for the CST of the human vaginal microbiome or opportunistic pathogens hidden in human microbiomes. For examples, in the case of AGP, Supplementary Table S5 (the first species listed) shows the minimum reads table for the most abundant OTU (*Bacteroidetes*_4468234). Figure 3 (also see Table 5) displayed the minimum sequencing reads (coverage) for monitoring (estimating) one of the CST indicator species of the HVM, *L. inners*, required for achieving various pre-specified precision levels, computed with single-species TPL-based optimum sampling formula.

As to the reason why either MSS (community sample scale) or MPS (single-species scale) is chosen as target of estimation for evaluating the quality of sequencing operation (the first application) or for determining the optimum sequencing reads is due to that only MSS or MPA is part of the *variance-mean* power-law model (TPL or TPLE) (Ma, 2015; Li and Ma, 2019a). We have tried to establish a similar power-law model with variance-richness or variance-diversity relationship, but failed. Although diversity-scaling with sampling units or the so-termed diversity–area relationship (DAR; Ma, 2018; Li and Ma, 2019b) or classic species–area relationship (SAR; Watson, 1835; Preston, 1960) does follow the power-law model, only power-law model that involves *variance* can be utilized to derive optimum sample size formula, which is obvious from Eq. 2.

Similar to the community-scale minimum reads table, parameter *b* of single-species TPL also strongly influences the change pattern of minimum reads. The change *trend* of minimum reads with the change of precision level (*P*) is independent of parameter *b*, but that with the change of MPS (*m*) is dependent on whether or not *b* > 3. If *b* < 3, the minimum reads decrease with the increase of *m*. But if *b* > *3*, the trend is opposite, that is, an increase in *m* leads to an increase in the minimum reads too. In existing literature, *b*-value is usually in the range between *1* and *2*, rarely exceeding *2*, not to mention of exceeding *3*. However, the case of *b* > 3 does exist in microbiome studies (Ma, 2015). Our interpretation for this phenomenon is as follows. The case of *b* > *3* indicates that the aggregation (single-species) or heterogeneity (community) is exceptionally high, which makes it harder to reliably detect those high-abundance patches and consequently needs to check more reads to achieve pre-specified precision.

## Conclusion and Discussion

Sequencing coverage is essentially a problem of sampling for quality control, and it is a mixture of science and art, because sampling is usually conducted with incomplete information. In other words, sampling or sequencing coverage problem has to deal with uncertainties. Therefore, coverage estimation cannot be perfect and there is no such a need either. Sampling is also inherently constrained by the need of being cost conscious. Determining the minimum sequencing efforts (i.e., optimum sequencing reads) for 16s-rRNA amplicon sequencing is essentially the same sampling problem that has been largely worked out in fields such as economic entomology and IPM. We borrow the TPL/TPLE-based optimum sample size formula from entomology to develop two categories of applications for amplicon sequencing. The first category of applications is based on the TPLE (Type-I or Type-II) (Ma, 2015) and can be utilized to (*i*) assess the quality of sequencing operation in hindsight, or (*ii*) estimate the *minimum* (*optimum*) *sequencing reads* (*coverage*) required to meet certain pre-specified precision and confidence levels, for planning a sequencing operation. The second category application is based on single-species TPL (Taylor, 1961, 1984), which is essentially the same as the traditional TPL-based approach widely adopted in economic entomology and IPM, and can be utilized to monitor single species-population abundances, such as the opportunistic pathogens or the indicator species for CSTs as defined by Ravel et al. (2011).

A contribution of this study is to identify and quantify the primary factors that can influence the *minimum sequencing efforts* required to satisfy desired relative precision level (*P*) and confidence level (the confidence level of 95% or Type-I error probability of α = 0.05) simultaneously. Sampling theory from elementary statistics, as demonstrated by Karandinos (1976) with Eq. 2, suggests that sampling variation (*V*) and mean (*m*) determines the optimum sample size when the precision and confidence levels are pre-specified. Taylor’s power law and its extensions establish the relationships between *V* and *m* at species and community scales, respectively (Eqs 1–3). The *V*–*m* power law not only makes the sampling biological populations somewhat unique, but also more convenient than sampling problems in other fields such as quality control of industry products. The approach has been widely adopted in many fields of agriculture, forestry, fishery, environmental sciences, and biomedicine. Some sampling procedures based on TPL have been established as national or international standards.

The power law based optimum sample size formula (Eq. 3) specifies a total of *5* parameters for estimating the *minimum sequencing reads* (coverage) in our case. Among the five parameters, error (precision) level (*D* or *P*) and confidence level (*t*_α_ = 2) parameters set the quality control parameters for determining *minimum sequencing reads*, and both of which should be set by decision-maker based on his or her project-specific objectives for microbiome sequencing. The values of TPL (TPLE) *a* and *b* are hopefully known for the decision-maker to determine the minimum sequencing reads based on the studies of the PL model parameters in existing literature. It was for this similar reason, in applied entomology, plant pathology and nematode and other relevant fields, the power law parameters for many economically important organisms are already known in existing scientific literature. We hope that in future, for the sake of evaluating the quality of sequencing coverage, power law analysis (Ma, 2015) is treated as a routine procedure in microbiome research. A fifth variable, *M* (the mean species size for TPLE, or mean population size for TPL) appears to be a “circular” parameter since the exact value of *m* can only be computed after the completion of the sequencing operation. However, in practice, a rough estimate of *M* based on the expertise of decision-maker can be used, which is also a standard practice in applied entomology and other relevant fields. For example, in a sequencing center, a rough estimation of *M* based on similar sequencing projects (such as gut microbiome studies) should be readily available. With the accumulation of more projects (experiences), a sequencing center may refine their estimations and establish standard operation procedures for each category of sequencing studies. Furthermore, the first application (approach) can be harnessed, in hindsight, to estimate the precision of completed projects. This feedback process can help further optimize established operational procedures, especially for a sequencing center.

The power law parameter *b* is species-specific (TPL) or community-specific (TPLE) characteristic. Hence, *b*-value can be utilized in cross platform settings. Nevertheless, the power law parameter *a* may be influenced by other sampling related factors such as sequencing platforms, primer used, etc. There may not be a perfect solution to deal with the uncertainty associated with the variability of parameter *a*. Nevertheless, sampling itself is an approximating or estimating process for the true values. The TPL (TPLE) based optimum sequencing reads may be a best educated guess we can achieve in many practical sequencing operations, which is obviously valuable despite certain level of controllable uncertainty. It is for this reason that sampling is considered as a mixture of art, science and drudgery (Wright and Palukkatu-Thodi, 2016). A promising measure to deal with this uncertainty associated with TPL (TPLE) parameter *a* is most likely still based on the TPL (TPLE). As demonstrated in sampling insect populations by Taylor (2018), abundance-dependent sampling efficiency can be estimated with TPL (Taylor, 2018). By establishing a reference sequencing platform such as we established for AGP and HVM in this study, and by comparing the *relative sampling efficiency* with the reference platform, one should be able to, at least partially, overcome the previously discussed uncertainties. It should be possible to borrow the principles and methods from successful solution demonstrated by Taylor (2018). Besides setting up reference sequencing platform, future studies designed for validating the proposed approach in this article should be performed. For example, comparative investigations with the coverage optimization approaches in metagenomics sequencing such as Lander–Waterman expectation theory and Stevens’ theorem could be invaluable.

## Data Availability Statement

The AGP dataset is available at: https://github.com/biocore/American-Gut/tree/master/data/AG. The HVMC dataset is available at: http://stm.sciencemag.org/content/4/132/132ra52.full.

## Author Contributions

ZM designed the study, interpreted the results and wrote the manuscript.

## Conflict of Interest

The author declares that the research was conducted in the absence of any commercial or financial relationships that could be construed as a potential conflict of interest.

## Footnotes

1 http://americangut.org/

2 https://github.com/biocore/American-Gut/tree/master/data/AG
