# Supplementary material for: Estimating the Optimum Coverage and Quality of Amplicon Sequencing With Taylor’s Power Law Extensions
Source: Front Bioeng Biotechnol. 2020 May 15;8:372. doi: 10.3389/fbioe.2020.00372 (PMC7242763; doi:10.3389/fbioe.2020.00372)
Supplement: Supplementary file 2 [file Data_Sheet_1.PDF]

Online Supplementary Information for:

Ma ZS (2020) Estimating the optimum coverage and quality of amplicon sequencing with Taylor's power law

List of Supplementary Tables

Table S1. The sequencing *precision* (quality) reversely (in hindsight) estimated with the TPLE-based optimum sample size formula for the 32-cohort HVMC study

Table S2. The minimal reads required for achieving certain precision levels estimated with Type-II-TPLE-based optimum sample size formula, illustrated with the subjects from 32-cohort HVMC study (Excel Table)

Table S3. The TPL (Taylor's Power Law) parameters for single-species populations of the AGP study

Table S4. The TPL (Taylor's Power Law) parameters for the single-species populations in the 32-cohort HVMC study

Table S5. The minimal sequencing efforts (reads) tables for each of the 10 selected species (selected from the AGP study) to achieve certain precision levels (***P***) for estimating the single-species population abundance based on the TPL-based optimum sampling formula.

Table S6. The minimal reads for the selected species (shared by no less than 20 subjects from 32-cohort study) required to achieve certain precision levels, estimated with TPL-based optimum sample size formula (Excel Table)

Table S7. The parameters of the Type-II TPLE models for 32-cohort HVMC study

Table S8. The TPL (Taylor's Power Law) parameters for the single-species populations: the top section displays the TPL parameters of 10 selected species built with the AGP dataset, and the full results for all species in the AGP dataset were exhibited in Table S3; the bottom section displays the TPL parameters of 10 selected species, built with the 32-HVM cohort datasets, and the full results for all species in the cohort were exhibited in Table S4.

Table S1. The sequencing *precision* (quality) reversely (in hindsight) estimated with the TPLE-based optimum sample size formula for the 32-cohort HVMC study

ID	*m*	*N*	*D (Error Level)*	(1-*D*) (Precision)
#400	43.383	2603	0.255	0.745
#401	27.563	2563	0.261	0.739
#402	25.777	2217	0.304	0.696
#403	85.296	2729	0.200	0.800
#404	28.002	2492	0.237	0.763
#405	59.138	2898	0.252	0.748
#406	35.172	2779	0.257	0.743
#407	22.718	2567	0.295	0.705
#408	44.144	2428	0.155	0.845
#410	39.832	2828	0.254	0.746
#411	28.124	2419	0.234	0.766
#412	72.859	2623	0.217	0.783
#413	18.509	870	0.298	0.702
#414	20.739	1099	0.230	0.770
#415	53.163	2977	0.232	0.768
#416	21.861	1377	0.339	0.661
#418	18.467	1293	0.422	0.578
#420	54.383	3480	0.213	0.787
#423	51.773	4970	0.229	0.771
#424	33.51	4122	0.252	0.748
#429	64.357	4762	0.226	0.774
#430	49.133	4127	0.104	0.896
#431	41.483	2365	0.174	0.826
#432	31.788	2797	0.298	0.702
#435	36.644	2272	0.250	0.750
#436	11.058	1979	0.250	0.750
#437	23.449	2134	0.376	0.624
#439	55.327	2324	0.181	0.819
#443	31.633	3448	0.228	0.772
#444	40.21	4302	0.154	0.846
#445	45.663	1827	0.194	0.806
#446	52.448	3042	0.236	0.764
**Average**	39.613	2710	0.244	0.756

Table S2 (Excel Table) The minimal reads required for achieving certain *precision* levels estimated with Type-II TPLE-based optimum sample size formula, illustrated with the subjects from 32-cohort HVMC study

Table S3 (Excel Table) The TPL (Taylor's Power Law) parameters for the single-species populations in the AGP study

Table S4. The TPL (Taylor's Power Law) parameters for the single-species populations in the 32-cohort HVMC study

Species	*b*	ln(*a*)	*CACD*	*R*	*p*	*n*	*Mean Population Abundance*
L.iners	1.559	2.329	0.015	0.951	0.000	32	974
L.crispatus	1.653	1.536	0.095	0.988	0.000	31	563.3
Atopobium	1.599	2.154	0.027	0.984	0.000	31	359.3
L.gasseri	1.735	1.848	0.081	0.979	0.000	27	96.7
Prevotella	1.670	2.203	0.037	0.975	0.000	32	89.7
Parvimonas	1.678	2.107	0.045	0.983	0.000	24	47.3
Sneathia	1.781	2.033	0.074	0.988	0.000	22	44.9
L.jensenii	1.696	2.047	0.053	0.989	0.000	22	43.7
Gardnerella	1.677	1.836	0.066	0.984	0.000	27	41.1
Peptoniphilus	1.643	2.164	0.035	0.981	0.000	32	37.1
Anaerococcus	1.856	1.892	0.110	0.987	0.000	31	31.2
Streptococcus	1.919	2.410	0.073	0.994	0.000	31	30.5
Corynebacterium	1.815	1.873	0.100	0.982	0.000	32	29.6
Ruminococcaceae.3	1.601	1.688	0.060	0.991	0.000	20	25
Mobiluncus	1.772	2.123	0.064	0.994	0.000	26	22.9
Aerococcus	1.637	2.041	0.041	0.987	0.000	28	17
Finegoldia	1.847	1.833	0.115	0.970	0.000	32	16.5
L.otu5	1.414	1.419	0.032	0.964	0.000	30	13.1
Megasphaera	1.736	2.391	0.039	0.995	0.000	20	11.7
L.otu4	1.666	1.452	0.113	0.979	0.000	24	10.8
Gemella	1.758	2.268	0.050	0.976	0.000	27	10.7
Peptostreptococcus	1.812	2.196	0.067	0.990	0.000	27	8.6
Lachnospiraceae.10	1.718	2.079	0.055	0.995	0.000	21	8
Lactobacillales.2	1.548	2.115	0.021	0.982	0.000	23	7.8
Staphylococcus	1.998	2.165	0.114	0.977	0.000	29	7.4
Veillonella	1.802	2.350	0.053	0.991	0.000	20	5.6
Escherichia.Shigella	1.959	2.933	0.047	0.993	0.000	20	5.6
Coriobacteriaceae.3	1.568	1.810	0.041	0.984	0.000	26	5.6
Dialister	1.563	1.628	0.056	0.959	0.000	31	4.4
Peptococcus	1.718	1.977	0.064	0.987	0.000	23	3.7
Allisonella	1.613	1.793	0.054	0.989	0.000	23	2.9
Porphyromonas	1.770	1.993	0.075	0.975	0.000	24	2.7
Ureaplasma	1.854	2.061	0.089	0.980	0.000	25	2.3
Campylobacter	1.605	1.870	0.046	0.991	0.000	26	2.3
Incertae_Sedis_XI.1	1.542	1.787	0.037	0.970	0.000	27	1.6
Incertae_Sedis_XI.2	1.588	1.931	0.037	0.976	0.000	23	1.5
Actinomyces	1.607	1.896	0.044	0.960	0.000	25	1.3
L.otu3	1.452	1.077	0.092	0.923	0.000	30	1.2
Facklamia	1.546	1.655	0.048	0.989	0.000	21	1.1
Arthrobacter	1.658	2.087	0.042	0.981	0.000	21	1.1
Enterococcus	1.893	2.619	0.053	0.979	0.000	20	0.9
Varibaculum	1.345	1.099	0.041	0.988	0.000	23	0.4
Segniliparus	1.474	1.538	0.039	0.969	0.000	22	0.3
Acinetobacter	1.611	2.087	0.033	0.966	0.000	20	0.1

Table S5. The minimal sequencing efforts (reads) tables for each of the 10 selected species (selected from the AGP study) to achieve certain precision levels (***P***) for estimating the single-species population abundance based on the TPL-based optimum sampling formula.

***Bacteroidetes*_4468234**											
m	***D****=0.01*	0.05	0.1	0.15	0.2	0.25	0.3	0.35	0.4	0.45	0.5
	P*=0.99*	0.95	0.9	0.85	0.8	0.75	0.7	0.65	0.6	0.55	0.5
1	1	1	1	1	1	1	1	1	1	1	1
50	189	8	2	1	1	1	1	1	1	1	1
100	486	19	5	2	1	1	1	1	1	1	1
200	1245	50	12	6	3	2	1	1	1	1	1
300	2160	86	22	10	5	3	2	2	1	1	1
400	3194	128	32	14	8	5	4	3	2	2	1
500	4325	173	43	19	11	7	5	4	3	2	2
1000	11091	444	111	49	28	18	12	9	7	5	4
1500	19241	770	192	86	48	31	21	16	12	10	8
2000	28444	1138	284	126	71	46	32	23	18	14	11
2500	38518	1541	385	171	96	62	43	31	24	19	15
3000	49346	1974	493	219	123	79	55	40	31	24	20
***Proteobacteria*_3944484**											
m	0.01	0.05	0.1	0.15	0.2	0.25	0.3	0.35	0.4	0.45	0.5
	0.99	0.95	0.9	0.85	0.8	0.75	0.7	0.65	0.6	0.55	0.5
1	369548	14782	3695	1642	924	591	411	302	231	182	148
2	483736	19349	4837	2150	1209	774	537	395	302	239	193
3	566255	22650	5663	2517	1416	906	629	462	354	280	227
4	633207	25328	6332	2814	1583	1013	704	517	396	313	253
5	690543	27622	6905	3069	1726	1105	767	564	432	341	276
6	741224	29649	7412	3294	1853	1186	824	605	463	366	296
7	786965	31479	7870	3498	1967	1259	874	642	492	389	315
8	828863	33155	8289	3684	2072	1326	921	677	518	409	332
9	867668	34707	8677	3856	2169	1388	964	708	542	428	347
10	903917	36157	9039	4017	2260	1446	1004	738	565	446	362
15	1058114	42325	10581	4703	2645	1693	1176	864	661	523	423
20	1183221	47329	11832	5259	2958	1893	1315	966	740	584	473
***Firmicutes*_4326080**											
m	0.01	0.05	0.1	0.15	0.2	0.25	0.3	0.35	0.4	0.45	0.5
	0.99	0.95	0.9	0.85	0.8	0.75	0.7	0.65	0.6	0.55	0.5
0.5	41579	1663	416	185	104	67	46	34	26	21	17
1.0	69437	2777	694	309	174	111	77	57	43	34	28
1.5	93727	3749	937	417	234	150	104	77	59	46	37
2.0	115958	4638	1160	515	290	186	129	95	72	57	46
2.5	136772	5471	1368	608	342	219	152	112	85	68	55
3.0	156523	6261	1565	696	391	250	174	128	98	77	63
3.5	175432	7017	1754	780	439	281	195	143	110	87	70
4.0	193648	7746	1936	861	484	310	215	158	121	96	77
4.5	211280	8451	2113	939	528	338	235	172	132	104	85
5.0	228408	9136	2284	1015	571	365	254	186	143	113	91
5.5	245095	9804	2451	1089	613	392	272	200	153	121	98
10.0	381438	15258	3814	1695	954	610	424	311	238	188	153
***Firmicutes*_4349946**											
m	0.01	0.05	0.1	0.15	0.2	0.25	0.3	0.35	0.4	0.45	0.5
	0.99	0.95	0.9	0.85	0.8	0.75	0.7	0.65	0.6	0.55	0.5
0.5	766071	30643	7661	3405	1915	1226	851	625	479	378	306
0.6	745786	29831	7458	3315	1864	1193	829	609	466	368	298

0.7	729055	29162	7291	3240	1823	1166	810	595	456	360	292
0.8	714865	28595	7149	3177	1787	1144	794	584	447	353	286
0.9	702579	28103	7026	3123	1756	1124	781	574	439	347	281
1.0	691767	27671	6918	3075	1729	1107	769	565	432	342	277
1.5	651690	26068	6517	2896	1629	1043	724	532	407	322	261
2.0	624671	24987	6247	2776	1562	999	694	510	390	308	250
3.0	588480	23539	5885	2615	1471	942	654	480	368	291	235
4.0	564082	22563	5641	2507	1410	903	627	460	353	279	226
5.0	545856	21834	5459	2426	1365	873	607	446	341	270	218
10.0	492912	19716	4929	2191	1232	789	548	402	308	243	197
***Firmicutes*_375106**											
m	0.01	0.05	0.1	0.15	0.2	0.25	0.3	0.35	0.4	0.45	0.5
	0.99	0.95	0.9	0.85	0.8	0.75	0.7	0.65	0.6	0.55	0.5
0.1	460255	18410	4603	2046	1151	736	511	376	288	227	184
0.2	437043	17482	4370	1942	1093	699	486	357	273	216	175
0.3	424012	16960	4240	1884	1060	678	471	346	265	209	170
0.4	415003	16600	4150	1844	1038	664	461	339	259	205	166
0.5	408146	16326	4081	1814	1020	653	453	333	255	202	163
0.6	402629	16105	4026	1789	1007	644	447	329	252	199	161
0.7	398022	15921	3980	1769	995	637	442	325	249	197	159
0.8	394074	15763	3941	1751	985	631	438	322	246	195	158
0.9	390624	15625	3906	1736	977	625	434	319	244	193	156
1	387563	15503	3876	1723	969	620	431	316	242	191	155
1.5	376007	15040	3760	1671	940	602	418	307	235	186	150
2	368018	14721	3680	1636	920	589	409	300	230	182	147
10	326352	13054	3264	1450	816	522	363	266	204	161	131
***Firmicutes*_1028501**											
m	0.01	0.05	0.1	0.15	0.2	0.25	0.3	0.35	0.4	0.45	0.5
	0.99	0.95	0.9	0.85	0.8	0.75	0.7	0.65	0.6	0.55	0.5
0.1	1237264	49491	12373	5499	3093	1980	1375	1010	773	611	495
0.2	1207643	48306	12076	5367	3019	1932	1342	986	755	596	483
0.3	1190646	47626	11906	5292	2977	1905	1323	972	744	588	476
0.4	1178732	47149	11787	5239	2947	1886	1310	962	737	582	471
0.5	1169572	46783	11696	5198	2924	1871	1300	955	731	578	468
0.6	1162141	46486	11621	5165	2905	1859	1291	949	726	574	465
0.7	1155895	46236	11559	5137	2890	1849	1284	944	722	571	462
0.8	1150512	46020	11505	5113	2876	1841	1278	939	719	568	460
0.9	1145784	45831	11458	5092	2864	1833	1273	935	716	566	458
1	1141572	45663	11416	5074	2854	1827	1268	932	713	564	457
1.5	1125504	45020	11255	5002	2814	1801	1251	919	703	556	450
2	1114242	44570	11142	4952	2786	1783	1238	910	696	550	446
10	1053280	42131	10533	4681	2633	1685	1170	860	658	520	421
***Firmicutes*_190220**											
m	0.01	0.05	0.1	0.15	0.2	0.25	0.3	0.35	0.4	0.45	0.5
	0.99	0.95	0.9	0.85	0.8	0.75	0.7	0.65	0.6	0.55	0.5
0.1	710127	28405	7101	3156	1775	1136	789	580	444	351	284
0.2	600468	24019	6005	2669	1501	961	667	490	375	297	240
0.3	544350	21774	5443	2419	1361	871	605	444	340	269	218
0.4	507743	20310	5077	2257	1269	812	564	414	317	251	203
0.5	481053	19242	4811	2138	1203	770	535	393	301	238	192
0.6	460291	18412	4603	2046	1151	736	511	376	288	227	184
0.7	443437	17737	4434	1971	1109	709	493	362	277	219	177
0.8	429337	17173	4293	1908	1073	687	477	350	268	212	172

0.9	417273	16691	4173	1855	1043	668	464	341	261	206	167
1	406768	16271	4068	1808	1017	651	452	332	254	201	163
1.5	368753	14750	3688	1639	922	590	410	301	230	182	148
2	343955	13758	3440	1529	860	550	382	281	215	170	138
10	233001	9320	2330	1036	583	373	259	190	146	115	93
***Firmicutes*_291147**											
m	0.01	0.05	0.1	0.15	0.2	0.25	0.3	0.35	0.4	0.45	0.5
	0.99	0.95	0.9	0.85	0.8	0.75	0.7	0.65	0.6	0.55	0.5
0.1	709604	28384	7096	3154	1774	1135	788	579	444	350	284
0.2	552059	22082	5521	2454	1380	883	613	451	345	273	221
0.3	476657	19066	4767	2118	1192	763	530	389	298	235	191
0.4	429491	17180	4295	1909	1074	687	477	351	268	212	172
0.5	396145	15846	3961	1761	990	634	440	323	248	196	158
0.6	370830	14833	3708	1648	927	593	412	303	232	183	148
0.7	350693	14028	3507	1559	877	561	390	286	219	173	140
0.8	334136	13365	3341	1485	835	535	371	273	209	165	134
0.9	320181	12807	3202	1423	800	512	356	261	200	158	128
1	308193	12328	3082	1370	770	493	342	252	193	152	123
1.5	266099	10644	2661	1183	665	426	296	217	166	131	106
2	239768	9591	2398	1066	599	384	266	196	150	118	96
10	133853	5354	1339	595	335	214	149	109	84	66	54
***Firmicutes*_210292**											
m	0.01	0.05	0.1	0.15	0.2	0.25	0.3	0.35	0.4	0.45	0.5
	0.99	0.95	0.9	0.85	0.8	0.75	0.7	0.65	0.6	0.55	0.5
0.1	1112160	44486	11122	4943	2780	1779	1236	908	695	549	445
0.2	900233	36009	9002	4001	2251	1440	1000	735	563	445	360
0.3	795515	31821	7955	3536	1989	1273	884	649	497	393	318
0.4	728690	29148	7287	3239	1822	1166	810	595	455	360	291
0.5	680748	27230	6807	3026	1702	1089	756	556	425	336	272
0.6	643927	25757	6439	2862	1610	1030	715	526	402	318	258
0.7	614353	24574	6144	2730	1536	983	683	502	384	303	246
0.8	589836	23593	5898	2621	1475	944	655	481	369	291	236
0.9	569023	22761	5690	2529	1423	910	632	465	356	281	228
1	551029	22041	5510	2449	1378	882	612	450	344	272	220
1.5	486931	19477	4869	2164	1217	779	541	397	304	240	195
2	446028	17841	4460	1982	1115	714	496	364	279	220	178
10	273012	10920	2730	1213	683	437	303	223	171	135	109
***Firmicutes*_195933**											
m	0.01	0.05	0.1	0.15	0.2	0.25	0.3	0.35	0.4	0.45	0.5
	0.99	0.95	0.9	0.85	0.8	0.75	0.7	0.65	0.6	0.55	0.5
0.1	929621	37185	9296	4132	2324	1487	1033	759	581	459	372
0.2	655793	26232	6558	2915	1639	1049	729	535	410	324	262
0.3	534714	21389	5347	2377	1337	856	594	437	334	264	214
0.4	462623	18505	4626	2056	1157	740	514	378	289	228	185
0.5	413469	16539	4135	1838	1034	662	459	338	258	204	165
0.6	377209	15088	3772	1676	943	604	419	308	236	186	151
0.7	349045	13962	3490	1551	873	558	388	285	218	172	140
0.8	326353	13054	3264	1450	816	522	363	266	204	161	131
0.9	307566	12303	3076	1367	769	492	342	251	192	152	123
1	291678	11667	2917	1296	729	467	324	238	182	144	117
1.5	237826	9513	2378	1057	595	381	264	194	149	117	95
2	205761	8230	2058	914	514	329	229	168	129	102	82
10	91517	3661	915	407	229	146	102	75	57	45	37

Table 6 (Excel Table) The minimal reads for the selected species (shared by no less than 20 subjects from 32-cohort study) required to achieve certain precision levels, estimated with TPL-based optimum sample size formula

Table S7. The parameters of the Type-II TPLE models for 32-cohort HVMC study

ID	*b*	ln(*a*)	*CACD*	*R*	*p*	*N**	*Average Species Population Size*
#400	2.773	0.833	0.625	0.776	0.000	29	43.383
#401	1.874	4.194	0.008	0.653	0.000	30	27.563
#402	1.811	4.549	0.004	0.651	0.000	31	25.777
#403	2.509	1.047	0.500	0.906	0.000	32	85.296
#404	3.513	-1.485	1.806	0.836	0.000	30	28.002
#405	2.224	2.916	0.092	0.976	0.000	31	59.138
#406	2.634	1.565	0.384	0.738	0.000	31	35.172
#407	2.946	1.070	0.577	0.679	0.000	28	22.718
#408	1.471	4.688	0.000	0.631	0.000	29	44.144
#410	3.336	-1.099	1.601	0.810	0.000	29	39.832
#411	1.224	6.087	0.000	0.372	0.047	29	28.124
#412	3.042	-1.040	1.664	0.884	0.000	28	72.859
#413	0.515	7.294	3370927	0.246	0.208	28	18.509
#414	1.984	2.725	0.063	0.851	0.000	32	20.739
#415	2.220	2.818	0.099	0.852	0.000	30	53.163
#416	2.098	3.372	0.046	0.968	0.000	28	21.861
#418	2.082	3.814	0.029	0.851	0.000	31	18.467
#420	1.820	4.400	0.005	0.868	0.000	28	54.383
#423	1.627	5.650	0.000	0.426	0.021	29	51.773
#424	1.728	5.140	0.001	0.536	0.003	28	33.51
#429	2.622	1.522	0.391	0.905	0.000	29	64.357
#430	1.915	2.744	0.050	0.912	0.000	27	49.133
#431	3.572	-2.975	3.180	0.794	0.000	27	41.483
#432	1.714	5.118	0.001	0.709	0.000	29	31.788
#435	3.209	-0.779	1.423	0.817	0.000	25	36.644
#436	3.781	-0.844	1.355	0.584	0.000	32	11.058
#437	2.292	3.405	0.072	0.924	0.000	33	23.449
#439	1.059	6.720	0.000	0.495	0.005	30	55.327
#443	2.083	3.519	0.039	0.839	0.000	28	31.633
#444	1.969	3.353	0.031	0.882	0.000	27	40.21
#445	2.594	0.569	0.700	0.769	0.000	30	45.663
#446	1.757	4.703	0.002	0.984	0.000	29	52.448

**N* is the number of times the individual subject was sampled.

Table S8. The TPL (Taylor's Power Law) parameters for the single-species populations: the top section displays the TPL parameters of 10 selected species built with the AGP dataset, and the full results for all species in the AGP dataset were exhibited in Table S3; the bottom section displays the TPL parameters of 10 selected species, built with the 32-HVM cohort datasets, and the full results for all species in the cohort were exhibited in Table S4.

**Species**	***b***	**ln(*a*)**	**PACD**	***R***	***p-value***	***n***	**Mean Population Abundance**
**AGP dataset**							
Bacteroidetes_4468234	3.359	-10.668	97.702	0.772	0.000	26	2635
Proteobacteria_3944484	2.388	2.223	0.203	0.988	0.000	26	10
Firmicutes_4326080	2.740	0.552	0.724	0.872	0.000	26	3
Firmicutes_4349946	1.853	2.850	0.037	0.956	0.000	26	1.5
Firmicutes_375106	1.925	2.271	0.087	0.909	0.000	26	0.8
Firmicutes_1028501	1.965	3.351	0.031	0.982	0.000	25	0.5
Firmicutes_190220	1.758	2.319	0.047	0.928	0.000	26	0.3
Firmicutes_291147	1.638	2.042	0.040	0.924	0.000	26	0.2
Firmicutes_210292	1.695	2.623	0.023	0.965	0.000	24	0.1
Firmicutes_195933	1.497	1.987	0.018	0.946	0.000	23	0.1
... ...	... ...	... ...	... ...	... ...	... ...	... ...	... ...
**32-cohort HVM datasets**							
L. iners	1.559	2.329	0.015	0.951	0.000	32	974.0
L. crispatus	1.653	1.536	0.095	0.988	0.000	31	563.3
Atopobium	1.599	2.154	0.027	0.984	0.000	31	359.3
L.gasseri	1.735	1.848	0.081	0.979	0.000	27	96.7
Prevotella	1.670	2.203	0.037	0.975	0.000	32	89.7
Parvimonas	1.678	2.107	0.045	0.983	0.000	24	47.3
Sneathia	1.781	2.033	0.074	0.988	0.000	22	44.9
L. jensenii	1.696	2.047	0.053	0.989	0.000	22	43.7
Gardnerella	1.677	1.836	0.066	0.984	0.000	27	41.1
Peptoniphilus	1.643	2.164	0.035	0.981	0.000	32	37.1
... ...	... ...	... ...	... ...	... ...	... ...	... ...	... ...
