# Supplementary material for: Estimating the Optimum Coverage and Quality of Amplicon Sequencing With Taylor’s Power Law Extensions
Source: Front Bioeng Biotechnol. 2020 May 15;8:372. doi: 10.3389/fbioe.2020.00372 (PMC7242763; doi:10.3389/fbioe.2020.00372)
Supplement: Supplementary file 3 [file Data_Sheet_2.ZIP › Optimum Sequencing Reads-OSI-Code/Help-file.docx]

**Help file for three R-Scripts:** PowerLaw.r, MinReads.r **&** Precision.r

PowerLaw.r

**Function**: Computing the TPLE (Taylor’s power law extension) parameters for their applications to the “optimum sequencing coverage” problem.

(1) Program installation: The R version used in this code is version R3.5.1. The R-script (PowerLaw.r) and its input folder “Input” should be located in the same folder.

(2) Input file

The input file is an OTU table, an MxN matrix with elements representing the number of OTU reads for each OTU in the community, separated by “\t” (Tab) symbol, where M is the number of community samples, and N is the number of OTUs. The first row lists the OTU names, and the first column lists sample IDs or names. The input folder may have one or multiple input files, for building one or multiple TPLE models.

(3) Usage

Copy the R-script (PowerLaw.r) and input folder “Input” to the same folder. Enter the following command under Linux shell prompt:

$ Rscript PowerLaw.r

(4) Output file

The output file is named “TPL-parameters.txt” in “Output” folder, which contains columns of *b*, ln(*a*), CACD, *R*, *p*-value, *N.* The first three are the parameters of TPLE; the last three are parameters indicating the goodness-of-fitting to the PLE model.

MinReads.r

**Function**: Computing the “minimum sequencing reads” (MSR) for designing a sequencing project

(1) Program installation: The R version used in this code is version R3.5.1.

(2) Usage

Enter the following command under Linux shell prompt:

$ Rscript MinReads.r b lna

b and lna are the parameters of TPLE model. When computing the minimum sequencing reads (MSRs), we set a series of tolerable error levels “D” (1-Precision) and a series of roughly estimated mean reads (per OTU or species) “M”. We set D as 0.01, 0.05, 0.1, 0.15, 0.2, 0.25, 0.3, 0.35, 0.4, 0.45, 0.5 and set M as 1, 5, 10, 20 ... 100.

To compute the MSRs under arbitrary “D” and “M”, use the following command under Linux shell prompt.

$ Rscript MinReads.r b lna D M

(3) Output file

The output file named “MinimumReads.txt” is a matrix with elements representing the predicted MSR (minimum sequencing reads) for each error level (D) with a preset mean reads (M), separated by “\t” (Tab) symbol. The first row lists a series tolerable error levels (D), and the first column lists a series of M (the roughly estimated preset mean species abundance).

Precision.r

**Function**: Estimating the precision of a sequencing project *in hindsight*

(1) Program installation: The R version used in this code is version R3.5.1.

(2) Usage

Enter the following command under Linux shell prompt:

$ Rscript Precision.r b lna M N

For example: Rscript Precision.r 1.874 4.194 28 2563

b and lna are parameters of TPLE model; M is mean reads (per species or OTU); N is the total reads in a community sample (average reads per microbiome sample).

(3) Output file

The output file is named “Precision.txt” in “Output” folder, which contains the columns of M, N, D, & P. M is the mean reads (per species or OTU), N is the total reads in a community (i.e., average number of reads per microbiome sample), D is the estimated error level of the project under evaluation, P (=1-D) is the precision.
